# The Role of Asymmetric Interactions on the Effect of Habitat Destruction in Mutualistic Networks

**DOI:** 10.1371/journal.pone.0021028

**Published:** 2011-06-15

**Authors:** Guillermo Abramson, Claudia A. Trejo Soto, Leonardo Oña

**Affiliations:** 1 Centro Atómico Bariloche and CONICET, Bariloche, Argentina; 2 Instituto Balseiro, Bariloche, Argentina; 3 Department of Evolutionary Genetics, Max Planck Institute for Evolutionary Anthropology, Leipzig, Germany; University of Zaragoza, Spain

## Abstract

Plant-pollinator mutualistic networks are asymmetric in their interactions: specialist plants are pollinated by generalist animals, while generalist plants are pollinated by a broad range involving specialists and generalists. It has been suggested that this asymmetric –or disassortative– assemblage could play an important role in determining the observed equal susceptibility of specialist and generalist plants under habitat destruction. At the core of the analysis of the phenomenon lies the observation that specialist plants, otherwise candidates to extinction, could cope with the disruption thanks to their interaction with a few generalist pollinators. We present a theoretical framework that supports this thesis. We analyze a dynamical model of a system of mutualistic plants and pollinators, subject to the destruction of their habitat. We analyze and compare two families of interaction topologies, ranging from highly assortative to highly disassortative ones, as well as real pollination networks. We found that several features observed in natural systems are predicted by the mathematical model. First, there is a tendency to increase the asymmetry of the network as a result of the extinctions. Second, an entropy measure of the differential susceptibility to extinction of specialist and generalist species show that they tend to balance when the network is disassortative. Finally, the disappearance of links in the network, as a result of extinctions, shows that specialist plants preserve more connections than the corresponding plants in an assortative system, enabling them to resist the disruption.

## Introduction

Habitat destruction is the major cause of species extinctions and a main driving force behind current biodiversity loss [1]–[3]. In the context of habitat fragmentation, one of the most actively studied processes is animal-mediated pollination, which is crucial for the sexual reproduction of flowering plants. The strength of the effect of fragmentation on pollination and on plant reproductive success shows a highly significant correlation, suggesting that one of the most important causes of reproductive impairment in fragmented habitats may be pollination limitation [4].

In the mutualistic interaction between plants and pollinators, plant species are typically considered generalists when pollinated by several or many animal species of different taxa, and specialists if pollinated by one or a few taxonomically related pollinators [5]–[8]. Most plant-pollinator mutualistic networks have shown to be highly asymmetrical in their topologies, with specialist plants being pollinated mostly by generalist pollinators, whereas generalists are pollinated by both specialists and generalists pollinators [9], [10].

Some ecological consequences of the asymmetry of the plant-pollinator mutualistic network have been studied. Using mathematical models it has been shown that the asymmetry of plant-pollinator networks differs from random networks in their response to habitat destruction. Networks with topologies present in real communities start to decay sooner than random communities, but persist for higher destruction levels. When the destruction level is above a given threshold the whole community collapses [11].

Besides, theoretical studies have suggested that habitat destruction would affect preferentially specialised plants, because they would not be able to counterbalance the loss of their few specific mutualist partners with other alternative pollinators [5], [7], [12]. Generalist plants, instead, should be more adaptable to the changes imposed by fragmentation on their pollinator assemblages because the absence of one or some of their pollinators could be compensated by other pollinators from their wide assemblages [13]. Contrary to these theoretical expectations, no significant difference was found in the mean effect on specialist and generalist plant species, both being equally negatively affected by habitat fragmentation [14], [15]. One explanation for the equal susceptibility of specialist and generalist plants to habitat destruction lies precisely on the asymmetric interaction. Because specialist plants interact mainly with generalist pollinators, they would be able to keep their few pollinators in fragmented habitats, and thus their reproduction would not be so severely impaired as previously thought. Generalist plants, which interact with both generalist and specialist pollinators, would tend to loose their specialist pollinator fraction from their assemblages and retain their generalist pollinators. Thus, a decrease in the remaining generalist pollinators population would therefore have equal effects on the two groups of plants [16].

Mathematical models differ from verbal theories in giving a precise connection between assumptions and conclusions. They are a key tool needed to illuminate how the network architecture influences species extinction or persistence [17]. In this work we constructed assortative and disassortative networks and analyzed the effect of habitat destruction in each case, focusing on the relative effect on specialist and generalist species. We found that the way in which species are interconnected determines in a great deal who gets extinct, and in which way the perturbation affects the balance of specialization. In accordance with the theory proposed by Ashworth et al. [16], we observed that in asymmetric (disassortative) networks, generalist plants loose their connections with specialist pollinators, but specialist plants loose by far much less connections than specialist ones in the symmetric (assortative) networks. Our results support the idea that network asymmetry explains the equal susceptibility of generalist and specialist plants to habitat disturbance.

## Analysis

### Interaction networks

The interaction network of mutualistic, as well as many other ecological systems, is characterized by a highly heterogeneous connectivity. There are species –the generalists– that interact with many partner species, others that interact with few –the specialists– and all the intermediate cases. Moreover, the partners of specialist species are usually generalists and not other specialists. In the terminology of network theory this behavior is called *assortativity by degree* –rather, *disassortativity*, in this case. The *degree* of a node is the number of its connections. Assortativity refers to the fact that similar nodes connect between themselves. The similarity can be any individual characteristic of the nodes, and the assortative behavior of the network can be defined with respect to it. The degree, being a quantitative property of the nodes, allows a precise quantitative characterization of the assortative behavior, and it is also the property of interest in the specialist vs. generalist characterization of ecological networks.

If the average degree of the neighbors is plotted against the degree of the corresponding nodes, assortative networks display a growing relation –low degree connecting to low degree, middle to middle, high to high degree–. If, on the contrary, the relation is decreasing, low degree nodes have high degree neighbors: such is the hallmark of a disassortative network. Typically these relations are power laws, and the exponent can be used as a measure of the assortativity. Positive exponents describe assortativity by degree, and negative ones correspond to disassortativity. Mathematically, the assortativity is precisely measured as a correlation coefficient defined by (see for example [18], or [19] chapter 7):
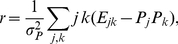
(1)which runs from 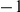 for completely disassortative behavior to 1 for completely assortative. Here 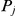 is the normalized distribution of the *remaining degree* of node 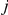 –the number of links leaving a node other than the one we arrived along–, 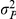 is its variance, and 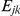 is the joint probability distribution of the remaining degrees of the two vertices at either end of a link.

As mentioned in the Introduction, it has been observed that mutualistic networks are *asymmetrical* in their connectivity, a fact that corresponds to the topological phenomenon of disassortativity in the theory of networks. Newman [18], indeed, has already observed that while social networks are generally assortative, biological and technological networks are disassortative. In what respects our present interest, the disassortativity of mutualistic networks has been proposed as the underlying reason for the equal susceptibility to the destruction of habitat of generalist and specialist plants [14]–[16]. In order to test the theoretical validity of this hypothesis we propose to analyse the dynamics of mutualistic systems based on several different models of interaction. We can manipulate these models in ways that cannot be done in natural systems, a fact that provides a good testbed for the construction of theoretical hypotheses and predictions.

**Figure 1:**
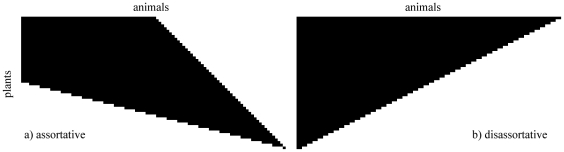
Assortative and disassortative interaction networks of bipartite systems. 50 plants (rows), 100 animals (columns). A black dot represents an interaction between the corresponding plant and animal. a) Assortative system (rhomboid model) with 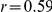; tends to connect generalist to generalist and specialist to specialist. b) The disassortative system (triangle model), 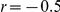, instead, tends to connect specialist to generalist. This last is the case sometimes called “asymmetric.”

Observe that the network of interactions that describes a mutualistic system is *bipartite*, i.e. there are links connecting only plants to animals and viceversa, but not plants to plants or animals to animals. Then, the relevant description of the interaction is given by a *biadjacency matrix*, defined as follows. Consider a matrix 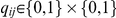, with 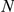 plants arranged as rows and 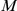 animals as columns. Matrix elements 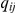 indicate the existence (1) or absence (0) of interaction between plant 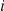 and animal 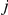. Figure 1 shows two extreme situations according to their assortativity, which we have termed the *rhomboid* and the *triangle* models. In Fig. 1(a) (a rhomboid) we see plants and animals connected in an assortative way: generalists to generalists, specialist to specialists. In contrast, Fig. 1(b) (a triangle) shows a system where plants and animals are connected disassortatively. The assortativity coefficients are 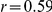 and 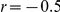 respectively. Regarding their assortative property, natural mutualistic systems are more similar to Fig. 1(b) than to 1(a). Natural mutualistic systems, also, have many less connections –we will come to this matter below. Let us first complete the formal definition of the models shown in Fig. 1, which will be used throughout this work. The *biadjacency matrix* 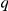, of the (bipartite) system in the rhomboid model is defined according to:
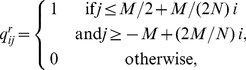
(2)which is a deltoid figure with its narrow angle pointing towards the specialists-specialists zone, shown in Fig. 1(a). The triangle model, in turn, is:
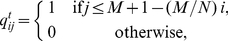
(3)as shown in Fig. 1(b) . Both models, as defined, have densities of links much higher than what is usually observed in nature. They can be easily diluted, by turning a fraction of 1's into 0's, to achieve any prescribed density. These networks will be kept constant during the course of our analysis, at variance with other analysis where the rewiring of links is allowed [20].

**Figure 2:**
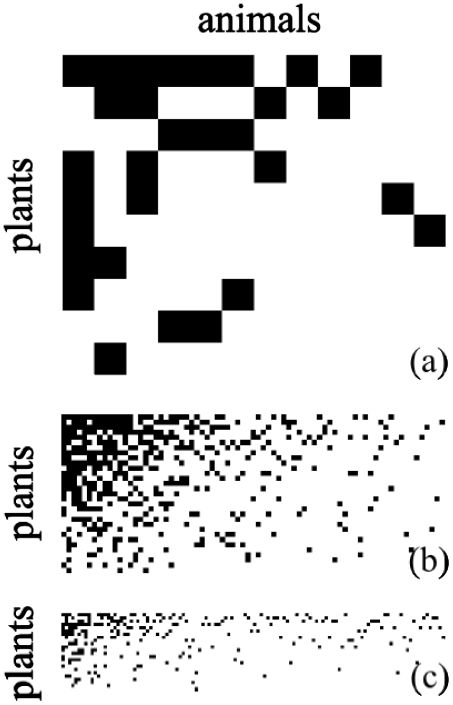
Interaction networks of mutualistic systems. Plants (rows) and animals (columns) are sorted from generalist to specialists (top to bottom and left to right respectively, as in Fig. 1). a) Flores Island (Azores archipelago), 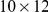, 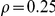, 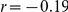. b) Zackenberg station (Greenland), 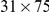, 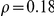, 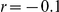. c) Abisko (northern Sweden), 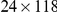, 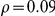, 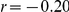. All three from [22].

**Figure 3:**
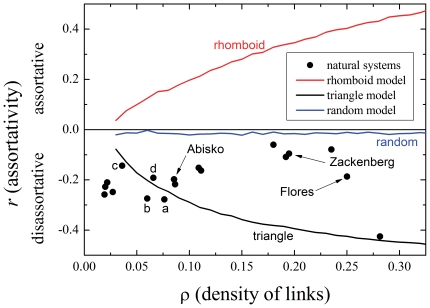
Assortativity as a function of density of links. The lines show the behavior of three models (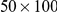 triangle, rhomboid and random, averaged of 100 realizations), while the points correspond to the real networks reported in [22]. The upper half of the plot correspond to assortative networks, while the lower half contains the disassortative ones (including all the natural systems). The density of links 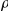 represents the number of links present in the system divided the total possible links (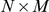). The networks represented in Fig. 2 are marked, as well as four other networks used below.

Figure 2 shows three examples of natural networks, taken from [21], [22]. We have ordered the species, plants as well as animals, according to their degree, from generalist to specialist. They display a feature that is common to many mutualistic systems: small systems tend to have a higher density links than larger ones. They also resemble the triangle model of Fig. 1(b), showing their disassortative topology. Lower density systems are less obvious under visual inspection, but the assortativity parameter, nevertheless, clearly characterizes them as disassortative (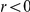). Figure 3 shows the relation between the assortativity 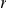 and the density of links 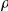 (defined as the number of links divided by the number of possible links) for all the pollination systems found in [21], [22] (circles). Along with these we show, also, families of varying dilution based on the models of Fig. 1. A random model (with links connecting pairs at random with uniform probability) is also shown for comparison: its assortativity is almost zero at all densities. (Actually, a random network is very slightly disassortative, as seen; this is a well known phenomenon of random graphs.)

It is clear that neither the triangle nor the rhomboid model represent *exactly* the complex architecture of a natural mutualistic system, not even in the behavior of collective parameters as those shown in Fig. 3. Indeed, it can be seen that the natural systems of medium and high density of links are *less* disassortative (greater 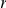) than the corresponding triangles of the same density. On the other hand, Fig. 3 also shows that the real systems of very low density are even *more disassortative* than the corresponding triangles. Both features, certainly, are due to the fact that natural networks are not built at random, but arise instead as the result of dynamics and evolution. We will address some of these questions later on.

One could, of course, define a linear combination of two models, a triangle and a rhomboid of a particular density, adequately weighted to give *any* intermediate value of the assortativity parameter. In such a way one could mimic the density and assortativity of any natural model in the intermediate and high density region. Networks with low density *and* very disassortative, however, cannot be represented by such a linear superposition. Other modelling choices are possible, though. For example, by starting from a random network of the right density of links, a Monte Carlo algorithm that interchanges the links of two pairs of nodes provides an easy way to modify the assortativity and achieve any desired value of 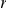, even very disassortative ones. We want to emphasize, though, that this is not what we pretend to do in this work. That is, we are not interested in modelling any particular real system with an artificial network. Instead, we are interested in the artificial networks as proxies for mutualistic systems of different assortativities. As already mentioned, by analyzing their dynamical behavior we pretend to test the hypothesis put forward by Ashworth et al. [16]: is the “asymmetry” of the network –characterized by its disassortativity– responsible for the similar susceptibility of generalists and specialists to habitat disturbance? Moreover, for each topological instance (say, for given 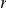 and 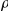) we can perform our analysis over a statistical ensemble of networks, and derive conclusions about their general behavior. The real networks, on the other hand, are singular. The triangle and rhomboid models are a good choice for this kind of analysis because their topological properties are simple and well separated from one another. Our *Gedankenexperiment* and our analysis will be based on their properties, and the analysis of the natural systems will be contrasted to theirs.

### Dynamical model of the mutualistic system

We study the population dynamics of the mutualistic system by means of a model based on the Levins model for metapopulations [23], [24]. The destruction of the habitat is modeled as in [25], with a single parameter 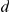. Let us say that there are 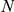 plants and 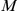 animals in the system, and let us call 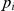 and 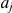 the population densities of plants and animals respectively. The evolution of these densities obeys the following dynamic equations (similarly to those proposed in [11]):
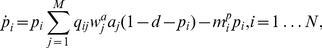
(4)

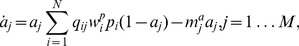
(5)where 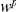 and 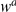 are interaction intensities for plants and animals respectively, while 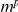 and 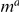 are death rates also for plants and animals. Let us discuss the interaction terms briefly, since there are several simplifications that we have preferred here, instead of more involved ones, in order to keep the number of parameters reasonably small. These simplifications allow us to concentrate on the effect of the network topology. Observe that both equations (4)–(5) are quadratic in the densities, of logistic type, without satiation factors. That is, plants can reproduce up to their carrying capacity as fast as it is allowed by the parameters of the model, as long as pollinators are present, without interference between the animals. Similarly, animals can reproduce proportionally to the plants density, disregarding any saturation at high plant density or any delay in the succession of generations. Observe also that the reproduction is obligatorily interspecific. The connectivity matrix 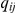 ensures that plant 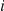 interacts with those animals 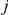 for which 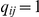 (remember that 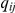 equals either 0 or 1, Eqs. (2) or (3)). The intensity of this interaction is proportional to the population of pollinator 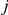, weighted by the parameter 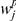 (which represents, for example, the rate of visits of pollinator 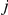 to plant 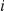). Each pollinator species contributes linearly to the whole reproduction rate. In a similar way, the reproduction of animal species 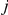 depends linearly, obligatorily, and weighted by 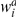, on the density of plant species 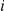.

With the purpose of keeping the analysis centered on the role of topology, we have chosen to use uniform distributions for the parameters 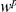, 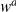, 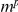 and 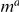 in the system. In this regard let us mention, nevertheless, that the intensities of interaction play an important role in the resistance to extinction. We have observed that if very small values of 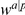 are allowed in the system, the fraction of extinct species is considerably large (because their contribution to reproduction is insufficient to allow stability). In these cases, the number of surviving species is too little to draw any significative statistical conclusions. To avoid these cases we have restricted the analysis, in what follows, to parameters 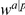 with an arbitrary value of 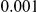 as a lower cutoff, so that both interactions are drawn from the interval 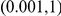 with uniform distribution.

Finally, the parameter that accounts for the destruction of the habitat is 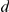, as mentioned, and we use it in our analysis as a control parameter of the dynamical system. Observe that it affects only the dynamics of the plants, reducing their carrying capacity in Eq. (4). With this, we are supposing that the destruction affects the available space for the sessile members of the community (through actual destruction, fragmentation, etc.), while the mobile pollinators are not directly affected by it. Of course, they feel the destruction indirectly by its effect on the plant populations.

The preparation of the initial condition of the system requires, beside the specification of the connectivity and the parameters, the specification of the initial densities of plants and animals. To mimic the conditions of a natural system one would aspire to have the dynamical system of Eqs. (4,5) at a steady state before subjecting it to a habitat perturbation by turning on the parameter 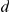. In our models we can achieve this by allowing a transient time for the system to evolve from an initial condition before starting our measurements. When using a random initial condition for the densities it is inevitable that some species evolve exponentially to zero, and get extinct during this transient, before the system reaches a stationary state. This transient, and these extinctions, of course, do not have any biological meaning. It is just a consequence of assembling a system of plants and their pollinators with random populations and random interactions between them. Natural systems are clearly not assembled in this way, but are the result of prolonged coevolution instead, from which a stationary state of coexistence arises. Our transient is only a procedure to achieve a similar stationary state, which we take as the initial condition of the system.

As a result of this random extinctions the network gets smaller, but we check that its size and its topological properties stay within a 10% of those initially defined. This, also, does not have any biological implication, and serves only the purpose of preparing the initial condition. All thse systems are taken together as replicas to perform statistical analysis.

For this system, then, a sudden destruction of a fraction of the habitat is simulated by setting a value of 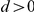. As a result of the perturbation, additional species get extinct until a new stationary situation is achieved. At this moment our simulation ends, and we proceed to measure the final properties of the system. The results reported below correspond to statistical averages over multiple initial conditions, network connectivities and intensities, as indicated in each case. A slightly different situation corresponds to the analysis of real networks, where the averages run only on initial conditions and parameters (but not on the network itself which is, naturally, singular).

### Extinctions in the perturbed system

**Figure 4:**
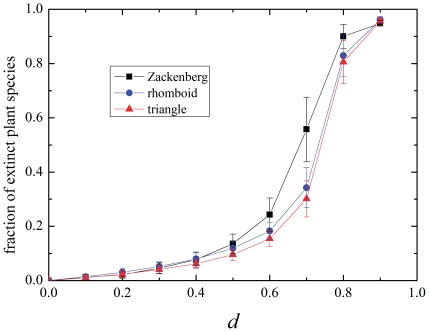
Fraction of extinct plants as a function of the destruction parameter, in two network models and a natural network. Links density is 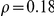, average of 1000 realizations.

Let us briefly explore the general behavior after the perturbation, before proceeding to the matter of extinctions by specialization degree. Figure 4 shows the fraction of extinct species of plants as a function of the destruction parameter 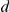. It is seen that a picture similar to the critical behavior explored in [26] arises: the extinction climbs steeply when a critical value of 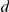 is approached. Since the size of the analyzed systems is finite the behavior is smooth, rather than abrupt as in a critical transition. Figure 4 shows a comparison between three networks of similar density: Zackenberg station, a triangle and a rhomboid. Observe how the real network is slightly more vulnerable than the artificial ones.

**Figure 5:**
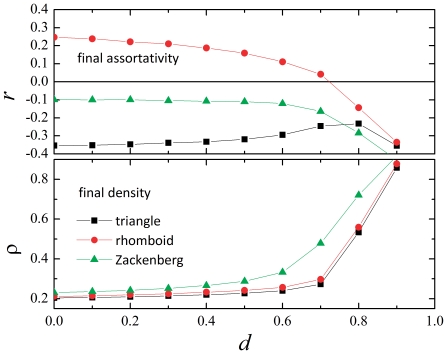
Final network properties as a function of the destruction parameter. Average of 1000 realizations for triangle and rhomboid models and for Zackenberg network. All with initial density of links 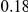. (Note that the density of links after the disruption is the number of existing links divided by the total number of possible links between the *extant* species.).

Besides the direct effect of erasing a fraction of plant and animal species, the disturbance has also an effect on the network. Indeed, when the system settles in its new equilibrium, the network of interaction and its topological properties have changed. Figure 5 quantifies this effect on the relevant parameters –assortativity and density of links–. Until 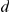 gets very high (above 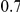, which implies a drastic modification of the system, as seen in Fig. 4), the density of links changes very little in both the disassortative and the assortative systems. The assortativity changes gradually when 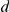 increases. It can be seen that the main modifications are suffered by the rhomboid network, which evolves towards a state of lower assortativity, until it turns disassortative at high 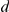. In other words, the dynamics of extinction drives the assortative network (the symmetric one) towards a disassortative state, a state of asymmetric interaction. The relevance of this fact on the *origin* of the observed asymmetric assemblage of natural mutualistic systems is without doubt an interesting one to be explored further, within a framework of evolving systems that lies beyond the present analysis.

### Differential extinction by specialization degree

In Fig. 4 we have seen that systems with very different topologies react in a similar way, regarding their loss of diversity (i.e., the fraction of extinct species), to the perturbation modeled as a destruction of habitat. In this section we show that, despite this global similarity, the way in which species are interconnected determines in a great deal *who* gets extinct, and how the perturbation affects the balance of specialization.

**Figure 6:**
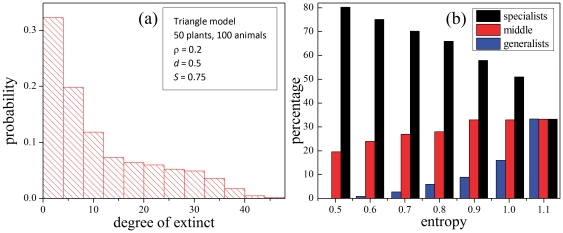
Entropy illustration. a) Distribution of the degree of the extinct species in a typical situation (1000 realizations). b) Typical distributions and the entropies that measure their degree of uniformity.

Following expectations, the degree of extinct species is always biased towards the specialists. The general situation is like the one shown in Fig. 6(a). This distribution corresponds to a triangle model network with density of links 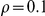, but in all regards it is representative of the general case. Beyond this general bias toward specialist species we want to quantify the differential effect of the extinction on generalist and specialist species. For this purpose we divide the totality of species (either plants or animals) in thirds according to their degree. We have three groups, then, and we call those most connected the *generalists*, those less connected the *specialists*, and the ones in the middle just so. The choice of just three groups is arbitrary, and has been preferred to having two groups in order to separate more clearly generalists and specialists. More than three groups are of course possible but, for our purposes, not much is gained in increasing the resolution in specialization degree.

Now, the question is how to measure the differential effect between generalists and specialists. Namely, how to distinguish between situations in which the extinct species belong more or less equally to the three classes (little advantage in being a generalist) from situations in which the extincts belong substantially to the specialist class? A reasonable measure of this effect is provided by an entropy, defined as follows. Suppose that 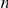 species get extinct at the end of the simulation, and that 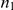 of them are specialists, 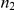 are in the middle, and 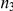 are generalists (so that 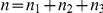). Define:
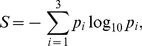
(6)where 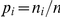. This differential entropy 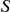 can range from 0 to 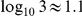. This highest value corresponds to a uniform distribution of extinctions in the three specialization classes. Lower values correspond to non-uniform distributions. Figure 6(b) shows a few distributions and the corresponding values of entropy, as an example. Note that the entropy defined by Eq. (6) is insensitive to which of the 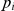 are greater and which smaller; it just measures their unbalance, disregarding if it is towards one class or another. This fact does not affect the analysis in the present case, since the distribution of extincts is *always* biased towards the specialists. Given this fact, the entropy provides a single parameter to quantify this bias, and one that can serve this purpose disregarding in how many specialization groups we divide the population.

**Figure 7:**
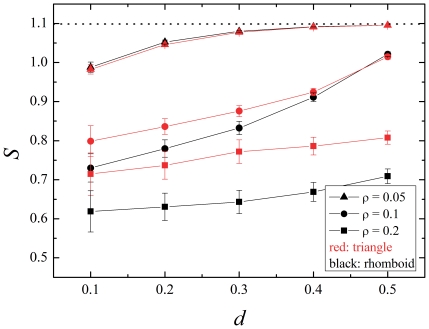
Entropies of the distribution of extincts, as a function of habitat destruction. Red: triangle model; black: rhomboid model. Three network densities are shown: low (▴), middle (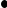) and high (▪). The dashed line near the top represents the maximum entropy, corresponding to a uniform distribution.

Figure 7 shows the entropy as a function of habitat destruction for several system topologies. Each point in the plot corresponds to the average of 1000 realizations. First, observe that the entropy grows with 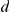. This means that greater habitat disruptions produce more uniform extinctions. Observe also that (for each symbol type, representing different network densities) the triangle model (disassortative) displays higher entropy than the rhomboid one (assortative). The three densities used also show that this effect is more pronounced in high density systems. This suggests a possible observation to be made in field studies: that in highly connected systems, asymmetric networks should display a balance (high entropy) in the resistance to extinction between generalists and specialists, while less asymmetric ones should show a preferential extinction of specialists (lower entropy).

On the other hand, it is seen that systems with low density appear close together and close to the null model (corresponding to a random elimination of species, without dynamical evolution, and which gives perfectly uniform distributions, 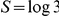 for all 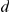).

**Figure 8:**
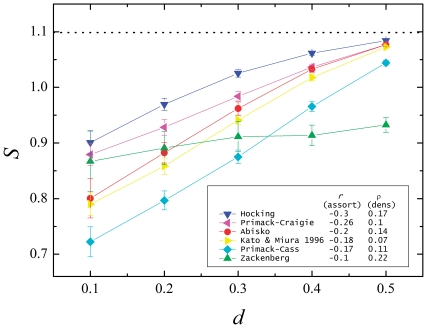
Entropies of the distribution of extincts vs. habitat destruction in natural networks. Several networks reported in [22] are shown, together with the values of their characteristic parameters *after the transient* (as explained in the section Dynamical model of the mutualistic system). Besides those already mentioned there are those marked with letters in Fig. 3: a) Hocking (arctic), b) Primack-Craigie (temperate), c) Kato & Miura 1996 (temperate), d) Primack-Cass (temperate). The dashed line near the top represents the maximum entropy, corresponding to a uniform distribution.

How do the natural networks compare with the behavior of the artificial models? Figure 8 shows a similar analysis performed on a set of real pollination networks from [22]. The Zackenberg station system stands out with a very flat dependence of the entropy 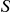 on the destruction 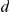 compared to the others. This network is the only one far from the low density cluster (see Fig. 3, dots marked a, b, c, d and Abisko, all of them close to the triangle model). In this regard its peculiar behavior is not surprising. Indeed, also a flatter dependence on 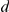 corresponds to the higher density models shown in Fig. 7. Nonetheless, the Zackenberg network has a higher entropy than the triangle with 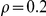 of Fig. 7. This is, naturally, consistent with the fact that the natural system is not randomly assembled. Its connectivity network is the result of its natural evolution (as its interaction parameters, discussed in the context of the initial conditions). As a consequence it shows a significant balance between generalists and specialists (along the lines of the field observations of [14], [15]). The other natural networks display a steeper dependence of the entropy on the destruction, also like the middle and low density models of Fig. 7. Bear in mind, however, that the dynamics mounted on the natural networks is simulated, and need not represent the dynamics of the real pollination systems; it is a dynamical property of their network that comes into view in the present analysis.

**Figure 9:**
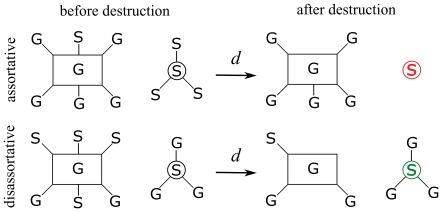
Protection of the links. Schematic representation of the protection against habitat destruction that generalist provide to specialist in disassortative (asymmetric) networks. (Adapted from Ashworth et al. [16]). In assortative networks the partners of specialists are other specialists. After the extinction of the most susceptible specialists their partners lose many or all their links, and remain extremely vulnerable (red). In disassortative networks, even when some species get extinct, the survivors retain some connection to the more robust generalists, therefore being protected (green).

To summarize, the entropy of the specialization degree of extinct species shows that, even if specialists are more susceptible to extinction, the distribution of extinct degrees is flatter in the triangle model (asymmetric, disassortative, closer to natural) than in the rhomboid model (symmetric, assortative, far from natural). In other words: generalist and specialist are more equally susceptible to extinction in disassortative networks. Recall the hypothesis of protection of specialists in disassortative networks, represented here as a diagram in Fig. 9 (Fig. 1 in [16]). The underlying idea is that the degree of the partners plays a crucial role: in asymmetric interactions, specialists are protected because they are connected to generalists. The degree of the neighbors is *precisely* what defines the assortativity of a network, so the behavior observed in our models supports that hypothesis.

**Figure 10:**
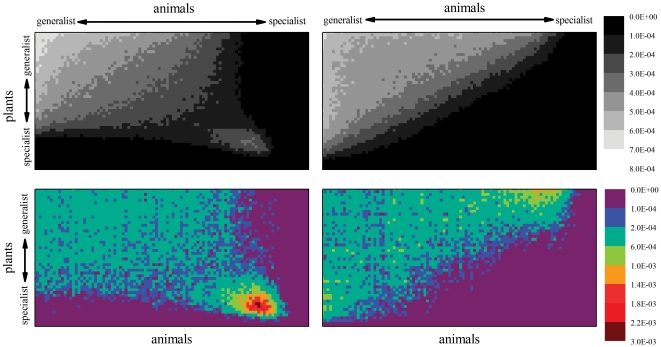
Removed links. Top: Probability density plots of existing links in assortative (left) and disassortative (right) models. Both cases correspond to 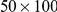 systems with a density of links 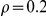, and are obtained from 1000 random realizations of the systems within the rhomboid and the triangle models. The probability is normalized for the whole network. Bottom: Probability density of the extinction of links when the parameter of destruction is 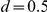, corresponding to the models on top. This probability is also normalized for the whole network. (The scales for the both plots of each row are shown on the right. Different shades are used to emphasize that the scales are different and that the represented magnitudes are different also.).

We can further test that assertion in the following way. Observe again Fig. 9 and consider not the extinct species but the *links* that disappear in the system when a species goes extinct. Now, in Fig. 10 the top two density plots show the probability distribution of the existence of links between a plant and an animal. This plots are related to the representations shown in Fig. 1 and 2, that correspond to single instances of the corresponding systems. The probability density is obtained from numerous realizations of systems with the same network parameters. The lighter colors show greater probability of connection between the corresponding plants an animal. Also in Fig. 10, the two bottom density plots show the corresponding probability of extinction of links when both models are subjected to a destruction 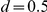. In these, the redder colors correspond to a higher probability that the corresponding link becomes extinct (also normalized to the whole network).

Observe, in Fig. 10, that in the rhomboid model the links with highest probability of disappearing are those connecting specialists to specialists. This region concentrates the majority of the broken links (21% in one ninth of the matrix corresponding to the specialist-specialist thirds). The situation corresponds to the one shown in Fig. 9 (top), where the specialist marked in red is left unconnected, and is a sure candidate for the next extinction. On the other hand, the assortative triangle model (bottom left in Fig. 10) shows that the probability of extinction of links is more evenly distributed. The links with highest probability of disappearance are certainly those belonging to specialists (each specialist-generalist ninth of the matrix harbors a 14% of the missing links). But, in this case, they are connected to generalists and do not further affect the system as much as in the assortative model. On the other hand, observe that the lack of the deeper shades of red (the highest values in the scale) show that the disappearing links are more evenly distributed. Their absence will affect the system evenly, with many specialists remaining protected by their connection to generalists, as shown in green in Fig. 9. This is precisely the phenomenon expected by the hypothesis put forward by Ashworth et al. [16].

## Discussion

Mathematical models of plant-pollinator interaction networks have given many insights into the effect of habitat fragmentation on ecological communities [11], [17]. One of the main characteristics of the topology of plant-pollinator interaction networks is their asymmetry: specialist plants are mainly pollinated by generalist pollinators whereas generalist plants are pollinated by both specialist and generalist pollinators [9], [10]. Such asymmetric interaction could be the reason why specialist and generalist plant species show similar response to habitat fragmentation, as argued in [16]. The main aim of this work has been to test this hypothesis while giving it a theoretical framework. To this goal, we have constructed symmetric and asymmetric networks of plant-pollinator interactions (Fig. 2). We have calculated the degree of asymmetry of such networks, as well as real ones, expressed by the measurements of their assortativity (Fig. 3). We then analyzed the extinction pattern of these networks as a function of the disturbance (Fig. 4). We have also analyzed the assortativity and density of the networks resulting from different degrees of habitat destruction (Fig. 5). We have introduced entropy as a measure of the differential effect of habitat fragmentation on generalist and specialist species (Fig. 6). Most importantly we have found that both the connectivity and the degree of habitat fragmentation are factors that increase the pattern of equal susceptibility of generalist and specialist plant species to habitat destruction (Fig. 7 and 8). A deeper analysis of the pattern of species extinction in symmetric and asymmetric networks shows that, in asymmetric (disassortative) networks, generalist plants loose their connections with specialist pollinators, but specialist plant loose by far much less connections than specialist plants in the symmetric (assortative) networks (Fig. 10). Therefore, and in accordance with Ashworth [16], our results suggest that network asymmetry explains the equal susceptibility of generalist and specialist plants to habitat disturbance.

Our approach is similar than the one from Fortuna [11] in that it does not include obligatory interactions on plants nor pollinators. We have assumed a community of facultative species in which the absence of their interacting partner does not implies species extinction. Obligatory interaction such as the one present in self-incompatible plants, may have a role on the pattern of species extinctions [4]. We did not include other complex features in our model such as temporal variation in the association plant-pollinator [27] or spatial effects [28]. These processes can have a role in the response to habitat destruction and deserve further investigation. Our aim was to capture, with the simplest model, the effect of asymmetry on the pattern of extinction in response to habitat destruction.
